# Effects of Copper and pH on the Growth and Physiology of *Desmodesmus* sp. AARLG074

**DOI:** 10.3390/metabo9050084

**Published:** 2019-04-30

**Authors:** Nattaphorn Buayam, Matthew P. Davey, Alison G. Smith, Chayakorn Pumas

**Affiliations:** 1 Master’s Degree Program in Applied Microbiology, Department of Biology, Faculty of Science, Chiang Mai University, Chiang Mai 50200, Thailand; nattaphorn.buayam@gmail.com; 2 Center of Excellence in Bioresources for Agriculture, Industry and Medicine, Department of Biology, Faculty of Science, Chiang Mai University, Chiang Mai 50200, Thailand; 3 Plant Metabolism Group, Department of Plant Sciences, University of Cambridge, Cambridge CB2 3EA, UK; as25@hermes.cam.ac.uk

**Keywords:** algae, copper, FT-IR, metabolite fingerprinting, pathway analysis, TEM

## Abstract

Copper (Cu) is a heavy metal that is widely used in industry and as such wastewater from mining or industrial operations can contain high levels of Cu. Some aquatic algal species can tolerate and bioaccumulate Cu and so could play a key role in bioremediating and recovering Cu from polluted waterways. One such species is the green alga *Desmodesmus* sp. AARLG074. The aim of this study was to determine how *Desmodesmus* is able to tolerate large alterations in its external Cu and pH environment. Specifically, we set out to measure the variations in the Cu removal efficiency, growth, ultrastructure, and cellular metabolite content in the algal cells that are associated with Cu exposure and acidity. The results showed that *Desmodesmus* could remove up to 80% of the copper presented in Jaworski’s medium after 30 min exposure. There was a decrease in the ability of Cu removal at pH 4 compared to pH 6 indicating both pH and Cu concentration affected the efficiency of Cu removal. Furthermore, Cu had an adverse effect on algal growth and caused ultrastructural changes. Metabolite fingerprinting (FT-IR and GC-MS) revealed that the polysaccharide and amino acid content were the main metabolites affected under acid and Cu exposure. Fructose, lactose and sorbose contents significantly decreased under both acidic and Cu conditions, whilst glycerol and melezitose contents significantly increased at pH 4. The pathway analysis showed that pH had the highest impact score on alanine, aspartate and glutamate metabolism whereas Cu had the highest impact on arginine and proline metabolism. Notably both Cu and pH had impact on glutathione and galactose metabolism.

## 1. Introduction

Copper (Cu) is widely used in industry [1] and as a result there is a very high demand of raw Cu extracted from mines. This demand is projected to increase 2.8 to 3.5 fold by 2050 [2]. Consequently, Cu containing waste (in particular wastewater) is generated daily from mining and industrial operations. The concentration of Cu in such mine drainage and water systems can vary substantially [3]. Additionally, the pH of the water systems is lowered (pH 3–5) resulting in acid mine drainage (AMD) [4,5]. A range of studies [3,6,7] have determined the level of Cu directly from industrial and acid mine run-off is around 10 to 80 times higher (up to 160 mg/L) than control level, indicating an urgent need for remediation. For example, the run-off from an abandoned Cu mine in Norway has a Cu concentration of 13.9 mg/L (pH 2.9), whereas the nearby receiving lake has just 0.04 mg Cu/L (pH 6.6) [3].

High levels of Cu exposure can be toxic to most living organisms [8,9,10,11] as heavy metals are non-biodegradable and environmentally persistent, which may be deposited on surfaces and then absorbed into the tissues of organisms [12]. Although the toxicity of Cu is recognized, the permissible amount of Cu in effluents slightly differs around the world. For example, the Ministry of Industry of Thailand has announced that the maximum permissible limit of Cu in industrial effluent is 2.0 mg Cu/L, which is the same as the permissible limit of Cu ions in drinking water permitted by the World Health Organization (WHO). The United States Environmental Protection Agency (USEPA) regulations state that Cu in industrial effluents must not exceed 1.3 mg Cu/L [1]. 

Despite the toxicity, some micro-algal species are able to grow in waterways that contain high concentrations of Cu, alongside the associated altered pH. This is in part due to Cu being an essential trace element that is required for normal algal growth [13,14] and also due to some algae (both macro- and micro-algae) being highly efficient in bioaccumulating heavy metals [15]. As such, bioaccumulation of the Cu from these polluted waterways is a potential approach for heavy metal bioremediation. Although the efficiency of heavy metal removal by algae and its effect on algal growth has been well characterized for some species [8,10,16,17,18,19] the biochemical mechanisms that are associated with algae tolerating high concentrations of Cu tolerance are not clearly defined or understood. In addition, *Desmodesmus* (Chlorophyta, Chlorophyceae) is the genera that frequently found distributed in the freshwater resources over the north and north-eastern of Thailand [20]. 

The aim of this study was to determine how microalgae are able to tolerate large alterations in their external Cu and pH environment. To this end we studied a green microalga *Desmodesmus* sp. (Chlorophyta, Chlorophyceae) AARLG074, a species isolated from and commonly found in a natural water reservoir in the north of Thailand. Specifically, we set out to measure the variations in the Cu removal efficiency, growth, ultrastructure, and cellular metabolite content in algal cells that are associated with Cu exposure and acidity. The Cu concentrations used in this study were set to the permittable Cu concentration in wastewater of Thailand (2 mg Cu/L) and Cu concentrations in the acid mine drainage system and industrial effluents (14–164 mg Cu/L) [3,6]. The pH values were also set according to the pH of AMD [4,5].

## 2. Results

### 2.1. Copper Removal Efficiency

The Cu removal efficiency of the cultures was assessed by growing *Desmodesmus* sp. AARLG074 in JM medium supplemented with 0, 2, 20, and 50 mg Cu/L at pH4 and pH6 for 168 h (7 days). The Cu removal efficiency was highest at pH6 where up to 83% of Cu in the media was absorbed within 30 min after inoculation (Table 1). The highest Cu absorption (93.5%) was measured 168 h after exposure to Cu in medium supplemented with 2 mg Cu/L, pH 6. Even in medium supplemented with 20 mg Cu/L the absorption after 24 h exposure at pH 6 was 92.8%.

In contrast, when algae were grown at pH 4 only 16.5% of Cu in the medium was removed at a Cu concentration of 20 mg Cu/L. The Cu absorption fluctuated over seven days with approximately 4–40% absorption at pH 4 and 64–94% at pH 6. Nonetheless, statistically significant lower Cu removal efficiency values were observed in all time points at pH 4, compared to pH 6 (Table 1).

### 2.2. Copper and Acid Exposure Affected the Cell Density, Pigment Content and Ultrastructure in Desmodesmus sp. AARLG074

**Figure 1:**
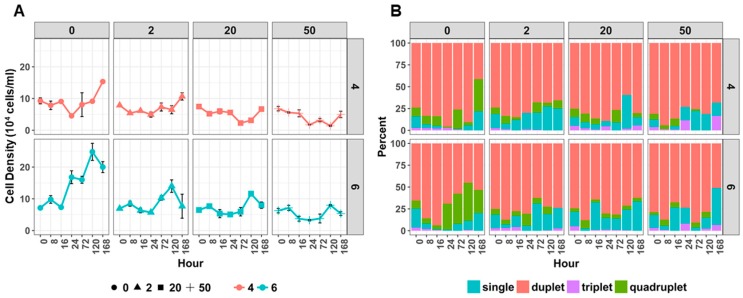
The effects of copper and pH on cell density and the percentage of number of cells per colony of *Desmodesmus* sp. AARLG074. (**A**) The mean cell density of *Desmodesmus* sp. AARLG074 grown at pH 4 (red line) and pH 6 (blue line) and copper concentrations (0, 2, 20, 50 mg/L) over 168 h. (**B**) The mean percentage of single (blue), duplet (pink), triplet (purple) and quadruplet (green) cells per colony of *Desmodesmus* sp. AARLG074 growing at pH 4 and pH 6 and copper concentration over 168 h. Values are mean ± SD (*n* = 3).

Cell and colony density—The cell density was calculated based on the number of colonies within four categories, based on number of cells in each colony (single, duplet, triplet, quadruplet) (Figure 1). The cell density of algae grown under control conditions (no Cu, pH 6) gradually increased and reached the highest cell density after 120 h cultivation (Figure 1A). However, the cell density of Cu-treated algae cultures at pH 6 was indicated a longer stationary phase (up to 72 h) with the highest cell densities occurring after 120 h cultivation. The cell density of algae grown in non-Cu supplemented JM at pH 4, which reflects that effects of acidic stress (pH 4) on algal growth, was significantly less than the cell densities of algae grown pH 6 (Figure 1A) (Supplementary Materials Table S1). As measured in cultures grown at pH 6, the cell density of cultures grown in combined Cu and pH 4 media also had a statistically-significantly negative effect on the cell density of *Desmodesmus* sp. AARLG074, when compared to no Cu media controls (Figure 1A).

The majority of colonies over the testing period were duplet (Figure 1B). The percentage of duplet colonies increased over 16 h cultivation under both control (0 mg Cu/L) and low Cu (2 mg Cu/L) conditions at pH 4 or pH 6. Additionally, the percentage of triplet colonies (three-cells-colony) significantly increased when exposed to high copper (50 mg Cu/L) for 168 h. The percentage of quadruplet colonies (four-cells-colony) also increased after 24 h cultivation, but this effect was negated when Cu was added to the media (Figure 1B and Supplementary Materials Tables S2–S5).

**Figure 2:**
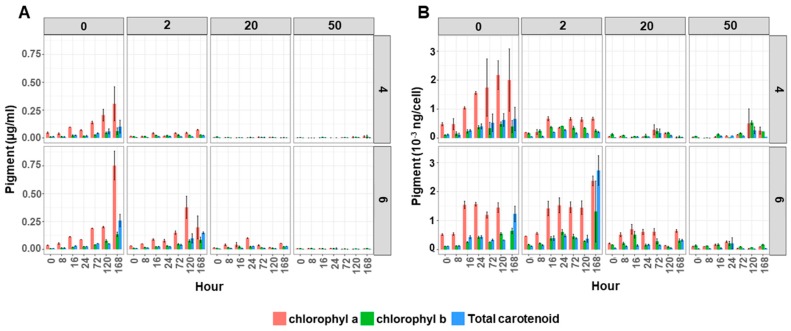
Pigment content (chlorophyll *a* (pink), chlorophyll *b* (green) and total carotenoids (blue)) of *Desmodesmus* sp. AARLG074 grown at pH 4 and pH 6 with varied copper concentrations (0, 2, 20, 50 mg/L) over 168 h. Data expressed as (**A**) pigments per volume of culture (ug per ml) and (**B**) per algal cell (ng per cell). Values are mean ± SD (*n* = 3).

Pigments—The pigment content (chlorophyll *a*, chlorophyll *b* and total carotenoids) of the cultures significantly decreased in the algal cultures containing Cu (Figure 2). This effect was more severe at pH 4 compared to pH 6 (Figure 2A and Supplementary Materials Tables S7–S9). The amount of pigments in the control (0 mg Cu/L) and low Cu (2 mg Cu/L) supplemented cultures gradually increased over time. This was not observed under high Cu supplemented conditions (20 and 50 mg Cu/L), where pigment concentrations were statistically lower than cultures grown at 0 and 2 mg Cu/L.

This lower pigment content in the culture would partly be due to the lower cell count per ml of culture, but also a lower pigment concentration per cell as there was a similar response to Cu and pH when the pigment values were expressed as amount of pigments per cell (Figure 2B and Supplementary Materials Tables S7–S9). The amount of pigments in un-supplemented Cu cultures grown in either pH 4 or 6 gradually increased over time (Figure 2B). Also, under low Cu supplemented conditions (2 mg Cu/L) the pigment content per cell increased until 16 h and remained stable until the end of experiment, in both pH 4 and 6. However, the amount of chlorophyll *a* in cultures grown at 50 mg Cu/L was significantly lower (Supplementary Materials Table S9). The amount of chlorophyll *a* in pH 4 grown cultures was lower than pH 6 but not statistically different.

**Figure 3:**
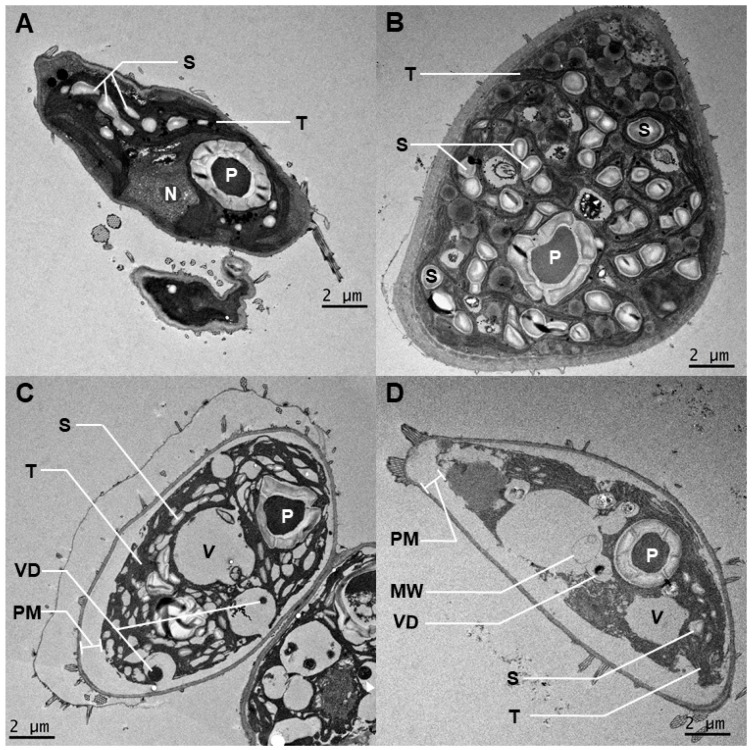
Electron micrographs of *Desmodesmus* sp. AARLG074 after 24 h growth in JM media at pH 4 and pH 6 at various Cu concentrations. The ultracellular structure of *Desmodesmus* sp. AARLG074 cultivated in the 0 mg Cu/L media at pH 4 (**A**) and pH 6 (**B**). Numerous starch granules (S) were observed. The ultrastructure of *Desmodesmus* sp. AARLG074 that grew in 50 mg Cu/L supplemented media at pH 4 (**C**) and pH 6 (**D**). Abbreviations: P, pyrenoids; PM, periplasmic space; S, starch granule; TH, thylakoids; V, vacuole; VD, vacuole deposit.

Ultrastructure—The ultrastructure of *Desmodesmus* sp. AARLG074 was altered after 24 h of Cu exposure as observed using TEM (Figure 3). Control algal cells that were grown in non-copper supplemented JM media at pH 4 had few starch granules with little or no periplasmic space (PM) (Figure 3A). However, control algal cells grown in non-copper supplemented JM media at pH 6 had many starch granules (S), again with little or no periplasmic space (PM) (Figure 3B).

In Cu supplemented media, the largest change in ultrastructure was observed in *Desmodesmus* sp. AARLG074 exposed to 50 mg Cu/L in both pH 4 (Figure 3C) and 6 (Figure 3D). The body of the cell had retracted from the cell wall resulting in an increased periplasmic space (PM) indicating that plasmolysis had occurred (Figure 3C,D). In addition, fewer starch grains (S) and membrane whorls (MW) were observed in the 50 mg Cu/L-treated cells at pH 6 (Figure 3D). The thylakoidal space was also more apparent under the high Cu and pH 4 (Figure 3C) conditions, compared to those grown under the high Cu and pH 6 conditions (Figure 3D).

### 2.3. The Effects of Copper and pH on Metabolite Composition of Desmodesmus sp. AARLG074

**Figure 4:**
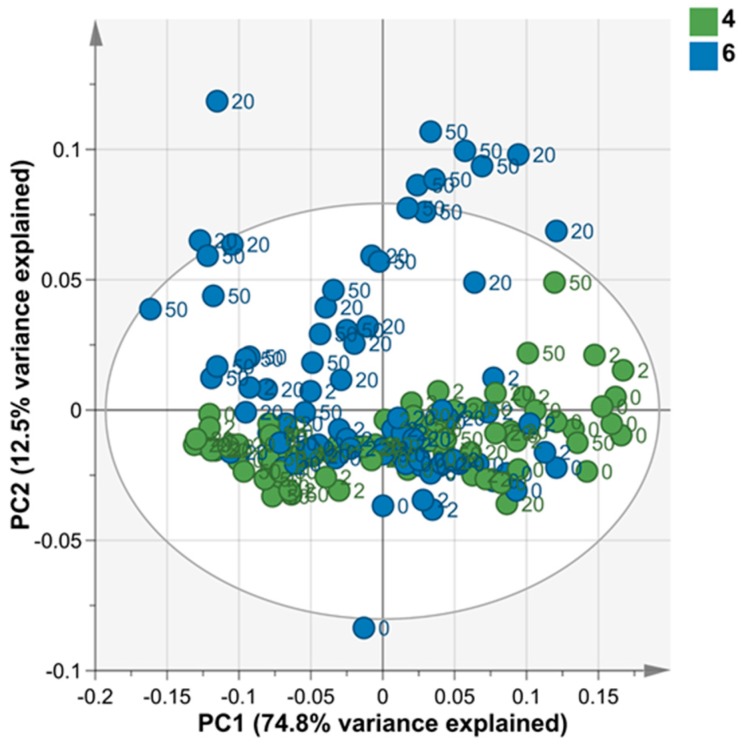
Score scatter plot from Principal Component Analysis (PCA) plot of FT-IR scans of *Desmodesmus* sp. AARLG074 cultured in JM under various copper concentrations (0, 2, 20 and 50 mg Cu/L) at pH 4 and pH 6. Each dot represents one biological replicate. The dot color represents the cultivation pH condition-pH 4 (green) and pH 6 (blue) and the labelled number beside dots represent the copper concentration as mg/L in the JM. The results shown here were analyzed using SIMCA-P (Version 15.0.2).

**Figure 5:**
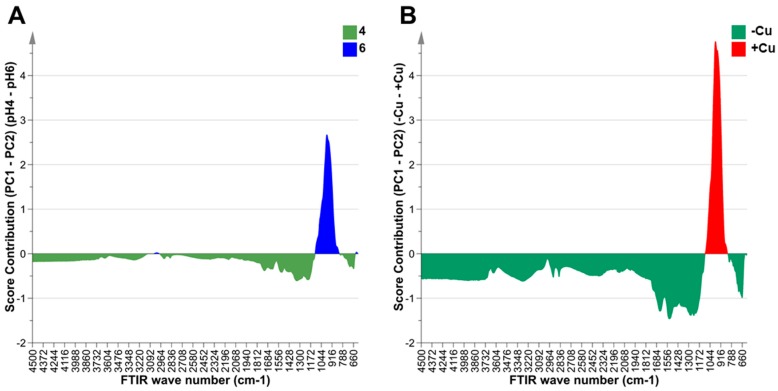
Score contribution plot values from PCA (PC1 and PC2 loading) from Fourier transform-infrared spectrometry (FT-IR) scans of *Desmodesmus* sp. AARLG074. FT-IR wavenumber score values are negative if they contribute towards PCA loadings associated with (**A**) growing in media at pH 4 (green) and positive for pH 6 (blue) and (**B**) negative if they contribute towards PCA loadings associated with no copper (green) and positive for copper supplemented media (red).

Fourier transform-infrared spectrometry (FT-IR) was used to study the effects of different Cu concentrations and pH conditions on the metabolite composition of *Desmodesmus* sp. AARLG074 over 168 h of cultivation. The results, based on the principal component analysis (PCA) (Figure 4), showed that the metabolite fingerprints of *Desmodesmus* sp. AARLG074 cultivated in high Cu (20 and 50 mg Cu/L) at pH 6 were different from the metabolite fingerprints of algae that were cultivated in any other conditions. The specific wavenumber regions that were strongly associated with high copper concentration treated *Desmodesmus* sp. AARLG074 at pH 6 only was in the range of 835–1090 cm^−1^ (Figure 5B), which was associated with wavenumbers relating to polysaccharides.

**Figure 6:**
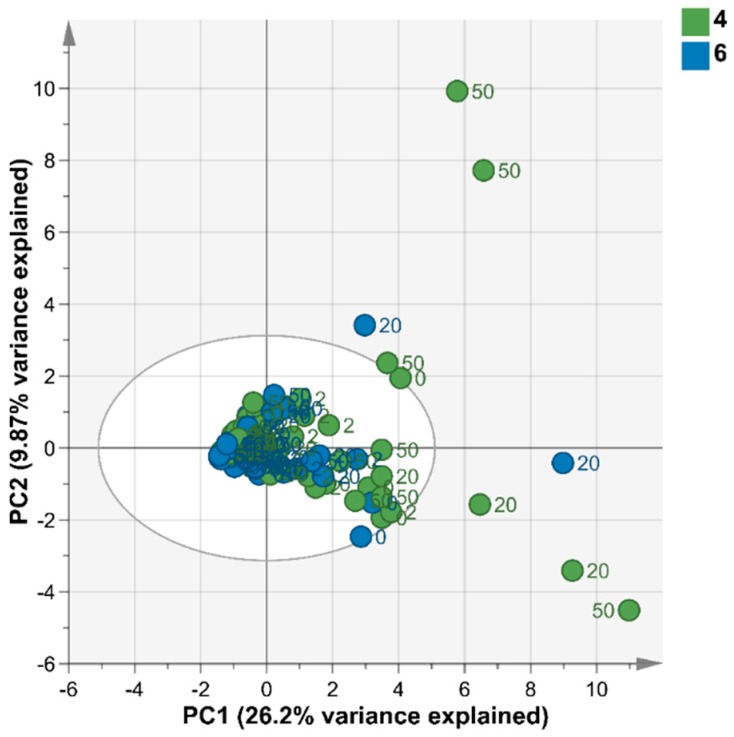
Metabolic profiling of the *Desmodesmus* sp. AARLG074 cultivated under different copper and pH conditions. The score scatter plot from PCA plot of GC-MS results of *Desmodesmus* sp. AARLG074 that were cultured in JM under various copper concentration (0, 2, 20 and 50 mg/L) at pH 4 and pH 6. Each dot represents one biological replicate. The colour of dot represents the cultivation pH condition-pH 4 (green) and pH 6 (blue) and the labelled number beside dots represent the copper concentration as mg/L in the JM.

**Figure 7:**
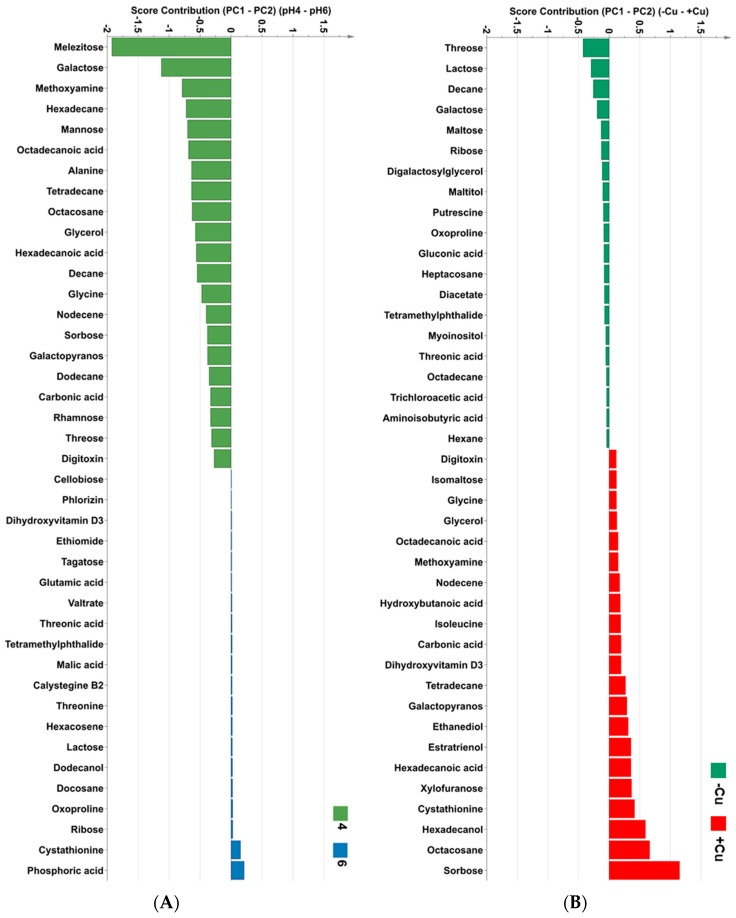
The score contribution plot values (top 40) that contribute towards GC-MS PCA loading plots of *Desmodesmus* sp. AARLG074 under different treatments (**A**) pH and (**B**) copper. The score contribution plot values (top 40) were ranked in order of importance and are negative if they contribute towards PCA loading plot for the pH 4 (green) and positive if they contribute towards the pH 6 (blue) and positive for copper stress (red) and negative if they contribute towards control (without copper) (green). The full list of the metabolites is presented in supplementary (Figures S1 and S2).

The detailed metabolite profiling by GC-MS did not reveal any major clustering of samples based on the pH and Cu concentration growth conditions in any of the principal components (Figure 6). Even though the PCA of the GC-MS data did not cluster by treatment, the score contribution plots of 40 key metabolites between absent Cu and Cu-supplemented treatment and between pH 4 and pH6 (Figure 7) were calculated to indicate the main metabolite differences between culture conditions. The score contribution of the full list of metabolites associated with the different Cu or pH are shown in Supplementary Materials Figures S1 and S2, respectively. The key metabolites based on the score contribution plots of both pH and Cu were polysaccharides, which corresponded to FT-IR results above (Figure 7). The other metabolites listed were lipids, amino and organic acids and vitamins. In pH 4 grown cells, the dominant metabolites were melezitose, galactose, hexadecane and mannose whereas in cells grown at pH 6 the dominant metabolites were phosphoric acid and cystathionine (Figure 7). The dominant metabolites in algae grown under high Cu supplemented media were sorbose, octacosane, hexadecanol, cystathionine and xylofuranose. The key metabolites under control conditions (zero Cu) were threose, lactose, galactose, maltose, ribose mannitol and lipids (e.g., decane and digalactosylglycerol). The statistical analysis of the detected metabolites showed that there were five statistically different metabolites among the treatments which were fructose, glycerol, lactose, melezitose and sorbose (Supplementary Materials Figure S3). Four of five statistically different metabolites among the treatments belonged to polysaccharides which supported the FT-IR results.

The impact of Cu and pH on the metabolic pathways (based on the number of metabolites per pathway being identified and changing in intensity using the GC-MS data) were analysed using the MetaboAnalyst Pathway-Analysis function [21]. The results which had *P* values less than 0.05 are shown in Table 2. The pathway analysis of different pH condition revealed that pH had impacted on multiple amino acid, sugar and lipid metabolic pathways. The greatest impact of pH was shown on alanine, aspartate and glutamate metabolism. Additionally, Cu exposure had also affected various protein metabolic pathways with the greatest impact on arginine and proline metabolism. Moreover, the result indicated that both pH and Cu had an effect on glutathione metabolism and galactose metabolism. Although the impact levels were low there were altered metabolic pathways related to photosynthesis- pigment synthesis and carbon fixation in photosynthesis. The results showed that seven amino acid or protein synthesis pathways had been affected by pH. However, only four amino acid synthesis pathways (arginine and proline metabolism, lysine biosynthesis, beta-alanine metabolism and butanoate metabolism) had been affected by Cu (Table 2).

## 3. Discussion

### 3.1. Tolerance of High Cu Exposure and Acidity in Desmodesmus sp. AARLG074—Growth and Structural Alterations Associated with Cu Removal

The aim of this study was to determine how *Desmodesmus* are able to physiologically tolerate large alterations in their external Cu and pH environment. *Desmodesmus* was indeed able to grow, although less so, in media supplemented with Cu. The Cu in the growth media was removed in the algae cultures with up to 83% of the copper present in the medium within 30 min after exposure, after which the absorption stabilizing over 168 h. We did not measure the Cu content in the cells but previous studies have shown that this biphasic absorption process may be due to a rapid non-metabolic dependent adsorption followed by a slow metabolic dependent uptake process [9,15]. The early absorption (<30 min) could be due to adsorption of Cu to the outer cell components (e.g., polysaccharides, mucilage and cell walls). The slower fluctuation in Cu removal efficiency (Table 1) would then be uptake into the living algal cell. In addition, the reduction in Cu removal efficiency at 20 mg Cu/L, pH 6 after 168 h exposure might be due to cell death and lysis, releasing the Cu back to the medium, or the living algae by export Cu out to the environment [15]. Moreover, the results also indicate that the pH is a critical factor that influences copper removal. The maximum removal, 93%, was when the algae were grown in JM media containing 2 mg Cu/L at pH 6. The efficiency of Cu absorption at pH 4 was significantly lower than pH 6, which was similar to several previous studies showing that heavy metal biosorption is a pH dependent process [22,23,24,25,26].

Despite the ability to tolerate Cu in the growth media, the exposure to Cu had an adverse effect on growth and cellular pigment content. Such effects have been previously observed and reported in other marine and freshwater algal species [8,10,16,17,18,19]. Although overall cell growth was reduced in cultures containing Cu we also found that the percentage of cell type was changed. There was a higher proportion of triplet cells in cultures that were cultivated in high Cu media at both pH 4 and pH 6 (Figure 1B). We can speculate that this might be one of the mechanisms that *Desmodesmus* sp. AARLG074 use for acclimating to live in a copper containing environment as multiple cell colonies have reduced cell surface area being exposed to the surrounding copper. Furthermore, the pigment analysis showed that chlorophyll *a*, *b* and total carotenoids content in algal cultures (Figure 2A) or per cell (Figure 2B), was lowered when cultivated in Cu supplemented media, this effect was much more severe in pH 4, a phenomena similar to previous studies [18,27,28,29]. Several studies have shown that the pigment level was reduced when algae were exposed to heavy metals [18,27,28,29], but none of them explain the reason of this phenomena. According to our TEM, ultrastructural changes of the algal cells occurred when exposed to Cu and acidity (Figure 3) notably at the high Cu concentration (50 mg Cu/L). One of the most drastic changes was the increase of intra-thylakoid space and disorganization of membrane which was similar to previous studies [19,24,29,30,31,32]. The alteration of the chloroplast ultrastructure was correlated with a lower chlorophyll content value (Figure 2). In addition, increasing the thylakoid space might be due to an excess of Cu accumulated inside due to Cu transporters locate on the pyrenoid and thylakoid membranes [33,34,35].

Furthermore, the Cu treated algae had fewer starch granules compared to the control, which might be due to an imbalance of the energy generated and used under Cu exposure as a consequence of chloroplast disorganization and fewer pigments [19,36]. Studies on another species of algae exposed to Cu, Desmidium swartzii, also had electron-dense inclusions in chloroplast and starch grains, of which Cu were detected in those compartments [37]. The presence of membrane whorls (MW) has previously been observed in heavy metal treated algae, where metals were detected inside the membrane whorl and as such has been proposed as a possible metal detoxification mechanism [38]

### 3.2. Metabolic Alterations Associated with Exposure to Cu and Acidity in Desmodesmus sp. AARLG074

The FT-IR results showed that different Cu and pH conditions affected a similar class of metabolites within the cells (Figure 4 and Figure 5). The wavenumbers that differentiated treatments were 835–1090 cm^−1^ (Figure 5A,B) which coincide with polysaccharide metabolites [39,40]. However, the GC-MS metabolite profiling showed that most of the key metabolites differed under the different pH or Cu conditions were sugars and amino acids (Figure 7). The PCA plots of both FT-IR metabolites fingerprinting and GC-MS metabolites profiling did indicate that there were relatively small differences in the metabolome of *Desmodesmus* sp. AARLG074 under various pH and copper treatments. This corresponds to a previous report on the effect of Cu on the transcriptome and metabolome of *Ectocarpus siliculosus* (Ochrophyta, Phaeophyceae) [41].

Five metabolites (glycerol, melezitose, lactose, fructose and sorbose) were highlighted as statistically different between treatments, which four of five are sugars (Figure S3) indicating some agreement between the FT-IR and GC-MS findings. From this list, the pH might be one of the crucial factors that affected the level of glycerol as there was considerably more glycerol in cells grown under pH 4 whereas there was no change under pH 6. The high level of glycerol at pH 4 might be explained for several reasons, largely osmoregulatory and membrane composition. Accumulation of intracellular glycerol in algae function as an osmoregulator which lowers internal osmotic potential and so prevent excessive water loss during high Cu exposure [42]. Additionally, released glycerol may also serve as a sink for products of photosynthesis [42]. Glycerol is also part of plasma membrane component and high glycerol induce membrane stiffening [43,44] and changes in membrane composition will affect the membrane permeability. The plasma membrane of algae living in acidic environments is fairly impermeable for protons, requiring relatively little energy for active pumping against a passive proton influx [45]. Thus, the higher level of glycerol detected in pH 4 might be one of the mechanisms that this green microalgae *Desmodesmus* sp. AARLG074 used to cope with the acidic and/or Cu stress. Moreover, melezitose (syn: melicitose), is an allelopathic chemical in marine planktonic microalgae [46]. This chemical is also found to be upregulated under osmotic stress in higher plants [47,48]. However, to our knowledge there is no previous report on an association between melezitose and Cu or pH in algae.

Beyond the top five metabolites mentioned above, one of metabolites that was highlighted in the score contribution plots as being important in cells grown in both pH 6 and Cu conditions was cystathionine (Figure 7). The cystathionine is an important intermediate compound in the biosynthesis of cysteine which is linked to glutathione metabolism [49]. The addition of glutamate to cysteine leads to form glutamyl cysteine, which the addition of glycine to this glutamyl cysteine leads to form glutathione [49]. This corresponded to our pathway analysis that showed Cu had impacted on glutathione metabolism (Table 2). Importantly, glutathione have been reported as an important intracellular ligands involved in metal sequestration and detoxification in algae [50].

Although the top five metabolites that were statistically different between the treatments were sugars the pathways that had the highest impact score for different pH and Cu were alanine, aspartate and glutamate metabolism (impact = 0.54) and arginine and proline metabolism (impact = 0.27) (Table 2). The pH treatment had the biggest impact on alanine, aspartate and glutamate metabolism, whereas Cu had the biggest impact on arginine and proline metabolism. The impact scores are a combination of the number of metabolites detected in the pathway and the fold change between treatments of those metabolites. This implies that although the metabolites may not statistically different between treatments, the impact score may still be on when taking all the metabolites in that pathway into consideration. Care should be taken when interpreting the in silico pathway analysis scores as the pathways were not measured directly, only the individual metabolites that could be in one or more metabolic pathway. However, such pathway analyses do provide us with an initial snapshot of which pathways were the most likely to be changing, which provides useful information for further targeted studies. There are similarities with the metabolites and pathways highlighted in the analysis in other organisms, especially bacteria [51,52,53,54,55]. Glutamate has been reported to be involved in acid tolerance in *Streptococcus mutans* [52], in both acid and Cu tolerance of *Escherichia coli* [53] and the accumulation of potassium glutamate in *E*. *coli* has been shown to occur immediately after osmotic shock to provide temporary protection to the cells [53,55]. In addition, aspartic acid and glutamic acid are also reported to be involved in acid tolerance in *Acetobacter pasteurianus* [51]. Proline is well known as playing an important role in plants and microorganism osmoprotection, metal chelation and general antioxidative defense molecule and signaling molecule [52,56,57]. Enhancement and accumulation of proline in heavy metals and acid exposed algae and bacteria has also been previously reported [48,57,58,59].

To conclude, we have shown that cultures of are able to tolerate and grow on media in both high Cu and acidic conditions (pH 4) and indeed removes over 80% Cu from the media within 30 min. The Cu concentration did affect Cu removal percentages. We showed that at the highest Cu removal efficiency and cell density, approximately 0.002 ng Cu could be removed per cell. This information shows that *Desmodesmus* sp. AARLG074 has the potential to industrially recover Cu from the algae that would otherwise be lost. However, despite its tolerance, the higher concentrations of Cu did negatively affect its growth rate. Additionally, we have shown that exposure and tolerance to Cu and acid conditions was associated with changes in its morphology, ultrastructure and overall metabolite composition of the cells, with polysaccharide and amino acid biochemistry changing the most.

## 4. Materials and Methods

### 4.1. Organism and Culture Conditions

The unicellular green microalga *Desmosdesmus* sp. AARLG074 was obtained from the Applied Algal Research Laboratory (AARL), Department of Biology, Faculty of Science, Chiang Mai University. This strain was isolated from the northern Thailand natural water reservoir [60]. The algal feed stock was maintained in Jaworski’s medium (JM), pH 7.0 under continuous white light emitting diode (LED) illumination (75 µmol photon/m^2^/s) at 25 °C and shaken by hand twice daily. The optical density 650 nm and cell density was measured until it reached the log phase after which the cultures were used as stock for further experiments. To study the effects of pH and Cu on Cu removal efficiency, algal growth, ultrastructure and metabolites the algae were inoculated into 1l flasks containing 800 mL Jaworski’s medium (JM) supplemented with 0, 2, 20 and 50 mg Cu/L by using CuSO_4_·5H_2_O as a Cu source. The pH of medium was adjusted to pH 4 and pH 6 using hydrochloric acid (HCl). Each treatment was conducted in triplicate biological samples (*n* = 3), in total 24 flasks were used. Atomic absorption spectroscopy (AAS) (see below method) confirmed the initial copper concentration in the treatment of 0, 2, 20, and 50 mg Cu/L was 0, 1.97 ± 0.11, 20.59 ± 0.55, and 50.66 ± 1.03 mg/L respectively.

### 4.2. Copper Absorption Efficiency

To determine the copper absorption efficiency, 10 mL of culture from three biological replicate samples (*n* = 3) was collected using an autopipette at various time points from 0 to 168 h (0, 0.5, 8, 16, 24, 72, 120 and 168 h) and then centrifuged at 3000 rpm for 5 min. The Cu concentration in the supernatant was measured using flame atomic absorption spectroscopy (AAS) as described in Mota et al. (2015) [61] and the removal efficiency (%) was calculated using the following equation:

Removal efficiency (%) = (100/Ci) × (Ci − Cf)(1)

where: Ci is the initial copper concentration (mg/L), Cf is the final (residual) copper concentration (mg/L).

### 4.3. The Effects of Copper and pH on Cell Density, Pigments and Ultrastructure

The three biological replicates of algae that were cultured in different conditions were collected at the same time point as described above. *Desmodesmus* sp. are colony forming. As 1, 2 and 4 cell colonies were observed (3 cell colonies were rarely found) the cell density (cells per ml of culture) was counted into four categories based on the number of cells per colony—single, duplet, triplet and quadruplet using a hematocytometer. The percentage of each type of colony found in the cultures was calculated to investigate the effect of pH and Cu on the change of percentage of each colony presented in algal community over time. Chlorophylls and carotenoids were extracted using 90% methanol as modified method from Saijo (1975) [62] and Lichtenthaler and Buschmann (2001) [63]. In addition, the amount of pigment per cell was calculated based on the amount of pigment in culture (µg/mL) and cell density (ng/cell). Transmission electron microscopy (TEM) was used to investigate the ultrastructural change of *Desmodesmus* sp. AARLG074 after 24 h exposed to Cu and acidic stress [38].

### 4.4. Metabolite Analysis

Ten ml of samples were collected at 0, 0.5, 8, 16, 24, 72, 120 and 168 h into the treatment phase and centrifuged at 3000 rpm at 4 °C for 5 min. The supernatant was discarded and the pellets were freeze-dried (freezone labconco, USA) and transferred to the Department of Plant Sciences, University of Cambridge, UK. Metabolite fingerprints of freeze-dried samples were obtained using a Perkin-Elmer Spectrum Two FT-IR, within the wavenumbers of 600–4500 cm^−1^. The spectra were normalized against air.

The metabolite profiling of freeze-dried samples was further investigated in more detail using GC-MS by extracting soluble polar and non-polar metabolites in methanol-chloroform-water method as described in Davey et al. (2005) [64]. The compounds within the polar methanol-water phase were derivatized by N-Methyl-N-trimethylsilyl-trifluoroacetamide (MSTFA) and Trimethylsilyl (TMS) as described by Dunn et al. (2011) [65]. Then, 1 μL (splitless) of the derivatized extracts were separated and profiled by GC-MS (GC-MS (Thermo Scientific Trace 1310 GC with 211 ISQ LT MS, Xcaliber v2.2) with a ZB-5MSi column (30 m, 0.25 mm ID, 0.25 μm film 212 thickness, Phenomenex, UK). GC-MS spectra were aligned to an internal standard (phenyl-β-d-glucopyranoside hydrate 98%) and processed using Thermo TraceFinder (v3.1) using genesis peak search method to aid identification based on molecular mass as previously described [64,66]. Pathway analysis of the identified metabolites were done both on SIMCA-P program (v15.0.2 Umetrics, Sweden) and MetaboAnalyst open source software (version 4.0, pathway analysis tool) using the *Arabidopsis thaliana* metabolic pathway library [21], www.metaboanalyst.ca). The results of pathway analysis that have *p* value ≤ 0.05 only were shown in the Table 2. R-script for the metaboanalyst software can be downloaded at https://github.com/xia-lab/MetaboAnalystR [21].

### 4.5. Statistical Analyses

To indicate the effect of initial Cu and pH on copper absorption efficiency, cell density and pigments of *Desmodesmus* sp. AARLG074 a two-way ANOVA (after a Lavene’s normality distribution) with Tukey HSD test (*p* ≤ 0.05) was performed using car, multcompView and lsmeans library on R version 3.4.3. as previously described [67]. Multivariate analyses to test whether the effect of initial Cu and pH on the algae could be discriminated based on their identified and unidentified metabolites (from FT-IR fingerprints or GC-MS profiling datasets) were performed using Principal Component Analysis (PCA) [68] on Pareto Scaled data (FT-IR absorbance values or GC-MS identified peak area units) within the SIMCA-P v14.1 PCA analysis pipeline (Umetrics, Sweden) to produce standard score scatter plots and ranked score contribution plots of how each variable (FT-IR wavenumber or GC-MS metabolite) contributed to clustering within the PCA score scatter plot.

## Figures and Tables

**Table 1:** Percent removal of copper from growth media containing *Desmodesmus* sp. AARLG074 over 168 h (7 days) growth at pH4 and pH6. Each value represents the mean ± SD (*n* = 3) with different superscript letter in the same time point indicating statistically significant differences (Two-way ANOVA, Tukey HSD, *p* < 0.05) using R version 3.4.3.

	Mean Percentage of Copper Removal from the Media ± SD (*n* = 3)					
pH	4			6		
Cu (mg/L)	2	20	50	2	20	50
0 h	0	0	0	0	0	0
0.5 h	20.3 ± 26.6 ^a^	13.1 ± 1.8 ^a^	17.7 ± 3.8 ^a^	57.6 ± 2.8 ^b^	82.6 ± 5.2 ^b^	82.5 ± 1.6 ^b^
8 h	27.6 ± 24.7 ^a^	15.5 ± 2.2 ^a^	13.0 ± 1.8 ^a^	67.2 ± 24.3 ^b^	82.6 ± 5.2 ^b^	81.0 ± 1.9 ^b^
16 h	4.1 ± 5.7 ^a^	14.0 ± 4.6 ^a^	14.6 ± 1.7 ^a^	64.6 ± 1.5 ^b^	88.9 ± 2.2 ^d^	77.2 ± 6.2 ^c^
24 h	10.7 ± 6.1 ^a^	16.5 ± 4.0 ^ab^	27.7 ± 9.1 ^b^	64.2 ± 1.4 ^c^	92.9 ± 4.0 ^d^	81.6 ± 2.6 ^d^
72 h	34.1 ± 19.4 ^a^	15.2 ± 4.9 ^a^	16.4 ± 2.9 ^a^	65.5 ± 0.5 ^b^	91.3 ± 2.1 ^c^	77.0 ± 5.0 ^bc^
120 h	40.6 ± 16.5 ^b^	18.0 ± 5.6 ^a^	15.8 ± 1.7 ^a^	64.6 ± 1.5 ^c^	83.7 ± 2.7 ^cd^	85.7 ± 1.2 ^d^
168 h	39.2 ± 18.9 ^a^	34.6 ± 0.6 ^a^	20.3 ± 0.6 ^a^	93.5 ± 1.2 ^b^	73.9 ± 1.1 ^b^	85.9 ± 0.1 ^b^

**Table 2:** Pathway analysis of the putatively identified metabolites by GC-MS in MetaboAnalyst open source 237 software (v4, pathway analysis tool) using the *Arabidopsis thaliana* metabolic 238 pathway library. The pathway analysis shows the effect of copper and pH on *Desmodesmus* sp. AARLG074 based on the number of metabolites matched in a metabolic pathway (Match), the statistical significance of the number of metabolites and their treatment response in that pathway (Raw P) and the importance of that pathway change in the network (Impact) [21].

Group of Metabolites	Pathway	pH			Copper		
		Match	Raw *P*	Impact	Match	Raw *P*	Impact
Lipid	Glycerolipid metabolism	3/13	2.86 × 10^−5^	0.08	-		
Amino acid	Glycine, serine and threonine metabolism	4/30	8.78 × 10^−3^	0.53	-		
	Cyanoamino acid metabolism	3/11	9.88 × 10^−3^	0.00	-		
	Selenoamino acid metabolism	2/19	3.98 × 10^−2^	0.00	-		
	Valine, leucine and isoleucine degradation	4/34	4.02 × 10^−2^	0.00	-		
	Alanine, aspartate and glutamate metabolism	4/22	4.04 × 10^−2^	0.54	-		
	Aminoacyl-tRNA biosynthesis	10/67	1.17 × 10^−2^	0.09	-		
	Nitrogen metabolism	3/15	2.06 × 10^−2^	0.00	-		
	Arginine and proline metabolism	-			5/38	2.15 × 10^−6^	0.27
	Lysine biosynthesis	-			1/10	1.40 × 10^−2^	0.00
	beta-Alanine metabolism	-			1/12	1.40 × 10^−2^	0.00
	Butanoate metabolism	-			2/18	4.01 × 10^−2^	0.00
Antioxidant or Homeostasis	Glutathione metabolism	4/26	1.20 × 10^−2^	0.09	4/26	2.84 × 10^−3^	0.09
	Ascorbate and aldarate metabolism	-			1/15	8.24 × 10^−3^	0.00
Carbohydrate (Sugar)	Galactose metabolism	5/26	1.65 × 10^−2^	0.09	5/26	3.03 × 10^−3^	0.09
	Inositol phosphate metabolism	-			1/24	8.24 × 10^−3^	0.25
Photosynthesis	Carbon fixation	2/21	3.94 × 10^−2^	0.00	-		
	Porphyrin and chlorophyll metabolism	-			1/29	4.32 × 10^−3^	0.00
Energy	Pentose phosphate pathway	-			2/18	7.60 × 10^−5^	0.00
	Glyoxylate and dicarboxylate metabolism	-			1/17	2.78 × 10^−3^	0.00
	Nicotinate and nicotinamide metabolism	-			1/12	1.40 × 10^−2^	0.00
	Methane metabolism	2/11	9.98 × 10^−3^	0.17	-		

## Supplementary Materials

The following are available online at https://www.mdpi.com/2218-1989/9/5/84/s1, Table S1: The effects of copper and pH over 7 days on cell density (cells/mL) of *Desmodesmus* sp. AARLG074, Table S2: The effects of copper and pH over 7 days on percentage of single colony (1-cell-colony) of *Desmodesmus* sp. AARLG074, Table S3: The effects of copper and pH over 7 days on percentage of duplet colony (two-cells-colony) of *Desmodesmus* sp. AARLG074, Table S4: The effects of copper and pH over 7 days on percentage of triplet colony (three-cells-colony) of *Desmodesmus* sp. AARLG074, Table S5: The effects of copper and pH over 7 days on percentage of quadruplet colony (four-cells-colony) of *Desmodesmus* sp. AARLG074, Table S6: The effects of copper and pH over 7 days on chlorophyll *a* (µg/mL) of *Desmodesmus* sp. AARLG074, Table S7: The effects of copper and pH over 7 days on chlorophyll *b* (µg/mL) of *Desmodesmus* sp. AARLG074, Table S8: The effects of copper and pH over 7 days on total carotenoids (µg/m) of *Desmodesmus* sp. AARLG074, Table S9: The effects of copper and pH over 7 days on chlorophyll *a* (10^−3^ ng/cell) of *Desmodesmus* sp. AARLG074, Table S10: The effects of copper and pH over 7 days on chlorophyll *b* (10^−3^ ng/cell) of *Desmodesmus* sp. AARLG074, Table S11: The effects of copper and pH over 7 days on total carotenoids (10^−3^ ng/cell) of *Desmodesmus* sp. AARLG074, Figure S1: Score contribution plot values showing the full list which contribute towards GC-MS PCA loading plots of *Desmodesmus* sp. AARLG074 under different pH condition, Figure S2: Score contribution plot values showing the full list which contribute towards GC-MS PCA loading plots of *Desmodesmus* sp. AARLG074 under different copper condition, Figure S3: Statistical analysis of GC-MS metabolite profiling of Desmodesmus sp. AARLG074 under copper and acidic stress.
