# Supplementary material for: Effects of Copper and pH on the Growth and Physiology of Desmodesmus sp. AARLG074
Source: Metabolites. 2019 Apr 30;9(5):84. doi: 10.3390/metabo9050084 (PMC6572535; doi:10.3390/metabo9050084)
Supplement: Supplementary file 1 [file metabolites-09-00084-s001.zip › Supplementary Materials/Supplementary Tables.docx]

**Supplementary Materials**

**Tables S1** The effects of copper and pH over 7 days on cell density (cells/ml) of *Desmodesmus* sp. AARLG074

|  | **Mean Cell Density ± SD (10^4^ cells per ml) (*n* = 3)** | | | | | | | |
| --- | --- | --- | --- | --- | --- | --- | --- | --- |
| **pH** | **4** | | | | **6** | | | |
| **Cu (mg/l)** | **0** | **2** | **20** | **50** | **0** | **2** | **20** | **50** |
| **0 hour** | 92,666 ± 9,451 ^c^ | 78,666 ± 3,055 ^bc^ | 74,666 ± 4,618 ^ab^ | 68,000 ± 6,928 ^ab^ | 71,333 ± 3,055 ^ab^ | 62,666 ± 7,023 ^ab^ | 78,666 ± 13,316 ^ab^ | 62,666 ± 7,023 ^a^ |
| **8 hours** | 78,666 ± 13,316 ^c^ | 54,000 ± 5,291 ^b^ | 52,000 ± 6,000 ^ab^ | 56,000 ± 2,000 ^ab^ | 98,000 ± 12,000 ^bc^ | 84,666 ± 8,082 ^ab^ | 76,666 ± 5,033 ^ab^ | 71,333 ± 8,082 ^a^ |
| **16 hours** | 90,666 ± 5,033 ^c^ | 61,333 ± 3,055 ^b^ | 60,000 ± 8,000 ^ab^ | 53,333 ± 11,015 ^ab^ | 73,333 ± 4,618 ^bc^ | 63,333 ± 7,571 ^b^ | 53,333 ± 12,858 ^ab^ | 37,333 ± 7,571 ^a^ |
| **24 hours** | 45,333 ± 1,154 ^bc^ | 49,333 ± 8,082 ^bc^ | 56,000 ± 2,000 ^c^ | 17,333 ± 1,154 ^a^ | 168,000 ± 20,297 ^d^ | 57,333 ± 3,055 ^c^ | 50,666 ± 5,033 ^bc^ | 32,666 ± 2,309 ^ab^ |
| **72 hours** | 80,666 ± 37,541 ^cd^ | 7,3333 ± 1,2701 ^bcd^ | 22,666 ± 3,055 ^a^ | 32,000 ± 5,291 ^ab^ | 160,000 ± 11,135 ^e^ | 102,666 ± 7,023 ^d^ | 59,333 ± 13,316 ^abcd^ | 38,000 ± 14,000 ^abc^ |
| **120 hours** | 91,333 ± 5,033 ^cd^ | 64,666 ± 12,701 ^bc^ | 31,333 ± 3,055 ^ab^ | 14,000 ± 2,000 ^a^ | 248,000 ± 26,907 ^f^ | 140,000 ± 19,697 ^e^ | 116,000 ± 4,000 ^de^ | 8,0000 ± 2,000 ^cd^ |
| **168 hours** | 153,333 ± 5,773 ^c^ | 106,666 ± 11,547 ^b^ | 66,666 ± 5,773 ^ab^ | 50,000 ± 10,000 ^a^ | 200,000 ± 17,320 ^d^ | 76,666 ± 37,859 ^ab^ | 80,000 ± 10,000 ^ab^ | 53,333 ± 5,773 ^a^ |

**Notes:** Each value represents the mean ± SD (*n=3*) with different superscript letter in the same time point indicate significant statistical differences (Two-way ANOVA, TukeyHSD, p<0.05) using R version 3.4.3

**Table S2** The effects of copper and pH over 7 days on percentage of single colony (1-cell-colony) of *Desmodesmus* sp. AARLG074

|  | **Mean Percentage of Single Cell per Colony ± SD (*n* = 3)** | | | | | | | |
| --- | --- | --- | --- | --- | --- | --- | --- | --- |
| **pH** | **4** | | | | **6** | | | |
| **Cu (mg/l)** | **0** | **2** | **20** | **50** | **0** | **2** | **20** | **50** |
| **0 hour** | 13.07 ± 2.66 ^a^ | 16.15 ± 2.01 ^ab^ | 9.91 ± 2.08 ^a^ | 8.55 ± 5.07 ^a^ | 21.51 ± 4.21 ^b^ | 15.41 ± 1.98 ^ab^ | 16.53 ± 2.05 ^ab^ | 14.90 ± 1.06 ^ab^ |
| **8 hours** | 4.22 ± 1.35 ^a^ | 6.31 ± 2.55 ^a^ | 6.78 ± 5.43 ^a^ | 0.00 ± 0.00 ^a^ | 6.16 ± 3.54 ^a^ | 3.98 ± 1.50 ^a^ | 2.62 ± 0.17 ^a^ | 4.61 ± 1.34 ^a^ |
| **16 hours** | 2.95 ± 1.30 ^a^ | 11.84 ± 4.65 ^abc^ | 5.94 ± 4.85 ^ab^ | 3.91 ± 4.41 ^ab^ | 0.88 ± 1.52 ^a^ | 12.64 ± 8.75 ^abc^ | 31.40 ± 12.99 ^c^ | 26.63 ± 14.61 ^bc^ |
| **24 hours** | 0.00 ± 0.00 ^a^ | 18.80 ± 2.02 ^d^ | 6.00 ± 2.21 ^abc^ | 15.28 ± 6.05 ^cd^ | 1.30 ± 1.33 ^ab^ | 5.76 ± 1.78 ^abc^ | 13.37 ± 3.54 ^bcd^ | 18.17 ± 9.77 ^cd^ |
| **72 hours** | 0.54 ± 0.93 ^a^ | 19.44 ± 3.06 ^bcd^ | 8.93 ± 1.16 ^abc^ | 22.11 ± 9.14 ^cd^ | 7.76 ± 6.17 ^abc^ | 31.22 ± 2.12 ^d^ | 13.04 ± 8.64 ^abc^ | 3.70 ± 6.42 ^ab^ |
| **120 hours** | 5.73 ± 3.08 ^a^ | 27.26 ± 20.33 ^ab^ | 38.55 ± 3.89 ^b^ | 18.65 ± 5.63 ^ab^ | 10.17 ± 5.31 ^a^ | 18.49 ± 11.64 ^ab^ | 23.52 ± 1.86 ^ab^ | 12.52 ± 6.64 ^ab^ |
| **168 hours** | 21.81 ± 4.26 ^a^ | 25.00 ± 5.00 ^a^ | 9.52 ± 8.25 ^a^ | 15.00 ± 13.23 ^a^ | 20.11 ± 5.10 ^a^ | 23.33 ± 8.82 ^a^ | 33.60 ± 14.56 ^a^ | 42.22 ± 23.41 ^a^ |

**Notes:** Each value represents the mean ± SD (*n=3*) with different superscript letter in the same time point indicate significant statistical differences (Two-way ANOVA, TukeyHSD, p<0.05) using R version 3.4.3

**Table S3** The effects of copper and pH over 7 days on percentage of duplet colony (two-cells-colony) of *Desmodesmus* sp. AARLG074

|  | **Mean Percentage of Duplet Cells per Colony ± SD (*n* = 3)** | | | | | | | |
| --- | --- | --- | --- | --- | --- | --- | --- | --- |
| **pH** | **4** | | | | **6** | | | |
| **Cu (mg/l)** | **0** | **2** | **20** | **50** | **0** | **2** | **20** | **50** |
| **0 hour** | 73.74 ± 5.90 ^ab^ | 73.74 ± 0.60 ^ab^ | 75.09 ± 2.64 ^ab^ | 80.98 ± 9.79 ^b^ | 65.44 ± 3.70 ^a^ | 75.00 ± 1.47 ^ab^ | 74.24 ± 0.74 ^ab^ | 78.66 ± 1.29 ^b^ |
| **8 hours** | 83.33 ± 5.81 ^ab^ | 83.50 ± 7.52 ^ab^ | 80.73 ± 3.41 ^a^ | 94.13 ± 3.87 ^b^ | 85.76 ± 3.62 ^ab^ | 87.29 ± 3.81 ^ab^ | 87.91 ± 2.22 ^ab^ | 87.23 ± 4.22 ^ab^ |
| **16 hours** | 83.74 ± 3.75 ^ab^ | 84.89 ± 4.53 ^ab^ | 86.33 ± 4.90 ^ab^ | 86.50 ± 4.23 ^ab^ | 93.76 ± 5.99 ^b^ | 77.58 ± 10.88 ^ab^ | 64.14 ± 17.23 ^a^ | 67.37 ± 9.65 ^a^ |
| **24 hours** | 95.59 ±0.11 ^c^ | 80.05 ±3.82 ^ab^ | 89.36 ±3.19 ^bc^ | 73.15 ±5.78 ^a^ | 68.85 ±7.50 ^a^ | 80.47 ± 7.30 ^abc^ | 80.13 ± 1.94 ^ab^ | 73.73 ± 8.01 ^a^ |
| **72 hours** | 76.11 ± 0.48 ^bcd^ | 67.93 ± 5.88 ^abc^ | 76.55 ± 3.65 ^bcd^ | 75.66 ± 6.31 ^bcd^ | 57.52 ± 3.85 ^a^ | 62.31 ± 2.19 ^ab^ | 81.39 ± 10.12 ^cd^ | 90.60 ± 4.71 ^d^ |
| **120 hours** | 90.61 ±2.83 ^c^ | 68.22 ± 17.35 ^b^ | 59.49 ± 2.74 ^ab^ | 81.35 ± 5.63 ^bc^ | 45.03 ± 4.41 ^a^ | 72.39 ± 10.71 ^bc^ | 71.26 ± 0.51 ^bc^ | 78.32 ± 2.92 ^bc^ |
| **168 hours** | 41.25 ± 7.01 ^a^ | 65.56 ± 5.09 ^ab^ | 80.16 ± 7.65 ^b^ | 68.33 ± 16.07 ^ab^ | 53.17 ± 3.64 ^ab^ | 73.89 ± 13.57 ^ab^ | 62.24 ± 21.66 ^ab^ | 51.11 ± 15.40 ^ab^ |

**Notes:** Each value represents the mean ± SD (*n=3*) with different superscript letter in the same time point indicate significant statistical differences (Two-way ANOVA, TukeyHSD, p<0.05) using R version 3.4.3

**Table S4** The effects of copper and pH over 7 days on percentage of triplet colony (three-cells-colony) of *Desmodesmus* sp. AARLG074

|  | **Mean Percentage of Triplets Cells per Colony ± SD (*n* = 3)** | | | | | | | |
| --- | --- | --- | --- | --- | --- | --- | --- | --- |
| **pH** | **4** | | | | **6** | | | |
| **Cu (mg/l)** | **0** | **2** | **20** | **50** | **0** | **2** | **20** | **50** |
| **0 hour** | 2.87 ± 1.15 ^a^ | 2.54 ± 0.10 ^a^ | 5.28 ± 2.37 ^a^ | 3.84 ± 1.23 ^a^ | 3.73 ± 1.58 ^a^ | 2.89 ± 0.09 ^a^ | 5.15 ± 1.78 ^a^ | 3.22 ± 0.36 ^a^ |
| **8 hours** | 2.59 ± 0.40 ^a^ | 1.39 ± 2.41 ^a^ | 2.43 ± 2.12 ^a^ | 1.23 ± 2.14 ^a^ | 2.06 ± 0.25 ^a^ | 3.20 ± 3.52 ^a^ | 0.00 ± 0.00 ^a^ | 1.04 ± 1.80 ^a^ |
| **16 hours** | 2.21 ± 0.12 ^ab^ | 0.00 ± 0.00 ^a^ | 0.98 ± 1.70 ^ab^ | 1.01 ± 1.75 ^ab^ | 0.88 ± 1.52 ^ab^ | 4.30 ± 2.08 ^b^ | 0.98 ± 1.70 ^ab^ | 0.00 ± 0.00 ^a^ |
| **24 hours** | 2.90 ± 2.51 ^ab^ | 1.15 ± 1.99 ^ab^ | 4.64 ± 5.25 ^ab^ | 11.57 ± 0.80 ^c^ | 0.00 ± 0.00 ^a^ | 0.00 ± 0.00 ^a^ | 1.33 ± 2.31 ^ab^ | 8.10 ± 3.19 ^bc^ |
| **72 hours** | 1.11 ± 1.92 ^a^ | 0.76 ± 1.31 ^a^ | 0.00 ± 0.00 ^a^ | 0.00 ± 0.00 ^a^ | 0.00 ± 0.00 ^a^ | 0.00 ± 0.00 ^a^ | 0.90 ± 1.56 ^a^ | 0.00 ± 0.00 ^a^ |
| **120 hours** | 0.00 ± 0.00 ^a^ | 0.00 ± 0.00 ^a^ | 1.96 ± 3.40 ^a^ | 0.00 ± 0.00 ^a^ | 1.13 ± 1.27 ^a^ | 0.43 ± 0.74 ^a^ | 0.60 ± 1.03 ^a^ | 2.48 ± 2.44 ^a^ |
| **168 hours** | 0.00 ± 0.00 ^a^ | 0.00 ± 0.00 ^a^ | 5.56 ± 9.62 ^a^ | 16.67 ± 28.87 ^a^ | 0.00 ± 0.00 ^a^ | 2.78 ± 4.81 ^a^ | 0.00 ± 0.00 ^a^ | 6.67 ± 11.55 ^a^ |

**Notes:** Each value represents the mean ± SD (*n=3*) with different superscript letter in the same time point indicate significant statistical differences (Two-way ANOVA, TukeyHSD, p<0.05) using R version 3.4.3

**Table S5** The effects of copper and pH over 7 days on percentage of quadruplet colony (four-cells-colony) of *Desmodesmus* sp. AARLG074

|  | **Mean Percentage of Quadruplet Cells per Colony ± SD (*n* = 3)** | | | | | | | |
| --- | --- | --- | --- | --- | --- | --- | --- | --- |
| **pH** | **4** | | | | **6** | | | |
| **Cu (mg/l)** | **0** | **2** | **20** | **50** | **0** | **2** | **20** | **50** |
| **0 hour** | 10.32 ± 3.74 ^b^ | 7.57 ± 2.25 ^ab^ | 9.72 ± 2.41 ^ab^ | 6.63 ± 3.72 ^ab^ | 9.32 ± 1.31 ^ab^ | 6.70 ± 1.42 ^ab^ | 4.08 ± 1.56 ^ab^ | 3.22 ± 0.36 ^a^ |
| **8 hours** | 9.86 ± 5.01 ^a^ | 8.81 ± 4.66 ^a^ | 10.06 ± 3.27 ^a^ | 4.64 ± 5.25 ^a^ | 6.02 ± 1.31 ^a^ | 5.53 ±1 .33 ^a^ | 9.47 ± 2.37 ^a^ | 7.11 ± 4.84 ^a^ |
| **16 hours** | 11.10 ± 2.54 ^a^ | 3.27 ± 0.17 ^a^ | 6.75 ± 0.91 ^a^ | 8.59 ± 8.34 ^a^ | 4.49 ± 2.95 ^a^ | 5.49 ± 4.22 ^a^ | 3.48 ± 3.08 ^a^ | 6.00 ± 5.89 ^a^ |
| **24 hours** | 1.52 ± 2.62 ^a^ | 0.00 ± 0.00 ^a^ | 0.00 ± 0.00 ^a^ | 0.00 ± 0.00 ^a^ | 29.85 ± 8.82 ^c^ | 13.77 ± 5.52 ^b^ | 5.16 ± 1.72 ^ab^ | 0.00 ± 0.00 ^a^ |
| **72 hours** | 22.24 ± 2.09 ^bc^ | 11.87 ± 7.59 ^ab^ | 14.52 ± 4.16 ^ab^ | 2.22 ± 3.85 ^a^ | 34.73 ± 8.78 ^c^ | 6.46 ± 3.92 ^a^ | 4.67 ± 2.26 ^a^ | 5.70 ± 1.78 ^a^ |
| **120 hours** | 3.66 ± 1.31 ^ab^ | 4.52 ± 3.02 ^abc^ | 0.00 ± 0.00 ^a^ | 0.00 ± 0.00 ^a^ | 43.67 ± 2.83 ^d^ | 8.69 ± 1.30 ^c^ | 4.62 ± 1.12 ^abc^ | 6.67 ± 1.46 ^bc^ |
| **168 hours** | 36.94 ± 3.37 ^b^ | 9.44 ± 10.05 ^a^ | 4.76 ± 8.25 ^a^ | 0.00 ± 0.00 ^a^ | 26.72 ± 7.20 ^b^ | 0.00 ± 0.00 ^a^ | 4.17 ± 7.22 ^a^ | 0.00 ± 0.00 ^a^ |

**Notes:** Each value represents the mean ± SD (*n=3*) with different superscript letter in the same time point indicate significant statistical differences (Two-way ANOVA, TukeyHSD, p<0.05) using R version 3.4.3

**Tables S6** The effects of copper and pH over 7 days on chlorophyll *a* (ug/ml) of *Desmodesmus* sp. AARLG074

|  | **Mean Chlorophyll *a* ± SD (*n* = 3)** | | | | | | | |
| --- | --- | --- | --- | --- | --- | --- | --- | --- |
| **pH** | **4** | | | | **6** | | | |
| **Cu (mg/l)** | **0** | **2** | **20** | **50** | **0** | **2** | **20** | **50** |
| **0 hour** | 0.047 ± 0.007 ^e^ | 0.015 ± 0.001 ^c^ | 0.004 ± 0.000 ^a^ | 0.002 ± 0.001 ^a^ | 0.037 ± 0.002 ^d^ | 0.032 ± 0.000 ^d^ | 0.014 ± 0.002 ^bc^ | 0.007 ± 0.000 ^ab^ |
| **8 hours** | 0.038 ± 0.008 ^b^ | 0.012 ± 0.005 ^a^ | 0.003 ± 0.001 ^a^ | 0.001 ± 0.0000 ^a^ | 0.052 ± 0.007 ^b^ | 0.050 ± 0.001 ^b^ | 0.041 ± 0.007 ^b^ | 0.008 ± 0.001 ^a^ |
| **16 hours** | 0.097± 0.001 ^cd^ | 0.042 ± 0.005 ^b^ | 0.001± 0.001^a^ | 0.002 ± 0.001^a^ | 0.113 ± 0.002 ^d^ | 0.089 ± 0.007 ^c^ | 0.042 ± 0.020 ^b^ | 0.007 ± 0.001 ^a^ |
| **24 hours** | 0.070 ± 0.005 ^b^ | 0.016± 0.001 ^a^ | 0.002 ± 0.000 ^a^ | 0.001± 0.000 ^a^ | 0.101 ± 0.003 ^bc^ | 0.088 ± 0.001 ^b^ | 0.077 ± 0.013 ^c^ | 0.009 ± 0.001 ^a^ |
| **72 hours** | 0.138 ± 0.012 ^d^ | 0.044 ± 0.003 ^c^ | 0.007 ± 0.003 ^ab^ | 0.003 ± 0.000 ^ab^ | 0.189 ± 0.002 ^e^ | 0.150 ± 0.016 ^d^ | 0.036 ± 0.004 ^bc^ | 0.002 ± 0.001 ^a^ |
| **120 hours** | 0.205 ± 0.053 ^b^ | 0.046 ± 0.005 ^a^ | 0.005 ± 0.001 ^a^ | 0.006 ± 0.006 ^a^ | 0.378 ± 0.100 ^b^ | 0.201 ± 0.007 ^c^ | 0.017 ± 0.001 ^a^ | 0.002 ± 0.001 ^a^ |
| **168 hours** | 0.307 ± 0.153 ^a^ | 0.073 ± 0.004 ^a^ | 0.002 ± 0.001 ^a^ | 0.012 ± 0.007 ^a^ | 0.753 ± 0.129 ^b^ | 0.197 ± 0.103 ^a^ | 0.054 ± 0.001 ^a^ | 0.005 ± 0.000 ^a^ |

**Notes:** Each value represents the mean ± SD (*n=3*) with different superscript letter in the same time point indicate significant statistical differences (Two-way ANOVA, TukeyHSD, p<0.05) using R version 3.4.3

**Tables S7** The effects of copper and pH over 7 days on chlorophyll *b* (ug/ml) of *Desmodesmus* sp. AARLG074

|  | **Mean Chlorophyll *b* ± SD (*n* = 3)** | | | | | | | |
| --- | --- | --- | --- | --- | --- | --- | --- | --- |
| **pH** | **4** | | | | **6** | | | |
| **Cu (mg/l)** | **0** | **2** | **20** | **50** | **0** | **2** | **20** | **50** |
| **0 hour** | 0.010 ± 0.002 ^bc^ | 0.013 ± 0.002 ^c^ | 0.010 ± 0.002 ^bc^ | 0.004 ± 0.000 ^a^ | 0.008 ± 0.000 ^ab^ | 0.012 ± 0.001 ^bc^ | 0.011 ± 0.000 ^bc^ | 0.009 ± 0.000 ^abc^ |
| **8 hours** | 0.012 ± 0.003 ^bcd^ | 0.014 ± 0.002 ^cd^ | 0.005 ± 0.000 ^ab^ | 0.0001 ± 0.000 ^a^ | 0.013 ± 0.002 ^cd^ | 0.018 ± 0.002 ^d^ | 0.016 ± 0.003 ^d^ | 0.009 ± 0.002 ^bc^ |
| **16 hours** | 0.022 ± 0.006 ^c^ | 0.023 ± 0.002 ^c^ | 0.003 ± 0.000 ^a^ | 0.006 ± 0.001 ^a^ | 0.020 ± 0.001 ^bc^ | 0.024 ± 0.001 ^c^ | 0.024 ± 0.007 ^c^ | 0.007 ± 0.003 ^ab^ |
| **24 hours** | 0.017 ± 0.002 ^bc^ | 0.021 ± 0.005 ^cd^ | 0.004 ± 0.003 ^ab^ | 0.001 ± 0.000 ^a^ | 0.024 ± 0.004 ^cd^ | 0.025 ± 0.002 ^d^ | 0.031 ± 0.004 ^cd^ | 0.007 ± 0.003 ^ab^ |
| **72 hours** | 0.026 ± 0.004 ^bc^ | 0.026 ± 0.006 ^b^ | 0.006 ± 0.001 ^a^ | 0.005 ± 0.000 ^a^ | 0.040 ± 0.002 ^cd^ | 0.046 ± 0.006 ^d^ | 0.017 ± 0.000 ^ab^ | 0.004 ± 0.0013 ^a^ |
| **120 hours** | 0.046 ± 0.008 ^b^ | 0.025 ± 0.001 ^ab^ | 0.006 ± 0.001 ^a^ | 0.007 ± 0.001 ^a^ | 0.076 ± 0.010 ^c^ | 0.076 ± 0.012 ^c^ | 0.011 ± 0.003 ^a^ | 0.006 ± 0.000 ^a^ |
| **168 hours** | 0.061 ± 0.030 ^ab^ | 0.028 ± 0.001 ^ab^ | 0.003 ± 0.002 ^a^ | 0.011 ± 0.013 ^a^ | 0.135 ± 0.020 ^c^ | 0.086 ± 0.025 ^bc^ | 0.024 ± 0.002 ^a^ | 0.009 ± 0.000 ^a^ |

**Notes:** Each value represents the mean ± SD (*n=3*) with different superscript letter in the same time point indicate significant statistical differences (Two-way ANOVA, TukeyHSD, p<0.05) using R version 3.4.3

**Tables S8** The effects of copper and pH over 7 days on total carotenoids (ug/m) of *Desmodesmus* sp. AARLG074

|  | **Mean Total Carotenoids ± SD (*n* = 3)** | | | | | | | |
| --- | --- | --- | --- | --- | --- | --- | --- | --- |
| **pH** | **4** | | | | **6** | | | |
| **Cu (mg/l)** | **0** | **2** | **20** | **50** | **0** | **2** | **20** | **50** |
| **0 hour** | 0.001 ± 0.002 ^e^ | 0.004 ± 0.000 ^c^ | 0.001 ± 0.000 ^ab^ | 0.0001 ± 0.000 ^a^ | 0.008 ± 0.000 ^d^ | 0.008 ± 0.001 ^d^ | 0.004 ± 0.001 ^bc^ | 0.002 ± 0.000 ^abc^ |
| **8 hours** | 0.009 ± 0.003 ^bc^ | 0.003 ± 0.001 ^ab^ | 0.001 ± 0.001 ^a^ | 0.0001 ± 0.000 ^a^ | 0.012 ± 0.003 ^c^ | 0.014 ± 0.001 ^c^ | 0.009 ± 0.002 ^bc^ | 0.002 ± 0.001 ^a^ |
| **16 hours** | 0.025 ± 0.002 ^c^ | 0.013 ± 0.001 ^b^ | 0.003 ± 0.001 ^a^ | 0.003 ± 0.000 ^a^ | 0.031 ± 0.002 ^d^ | 0.025 ± 0.002 ^c^ | 0.009 ± 0.004 ^ab^ | 0.002 ± 0.001 ^a^ |
| **24 hours** | 0.018 ± 0.004 ^cd^ | 0.014 ± 0.002 ^bc^ | 0.003 ± 0.000 ^a^ | 0.001 ± 0.000 ^a^ | 0.028 ± 0.001 ^de^ | 0.025 ± 0.002 ^de^ | 0.024 ± 0.002 ^e^ | 0.007 ± 0.007 ^ab^ |
| **72 hours** | 0.042 ± 0.004 ^bc^ | 0.011 ± 0.001 ^a^ | 0.004 ± 0.003 ^a^ | 0.002 ± 0.000 ^a^ | 0.053 ± 0.000 ^c^ | 0.040 ± 0.006 ^b^ | 0.009 ± 0.002 ^a^ | 0.001 ± 0.001 ^a^ |
| **120 hours** | 0.059 ± 0.022 ^bc^ | 0.012 ± 0.000 ^ab^ | 0.003 ± 0.000 ^ab^ | 0.004 ± 0.001 ^ab^ | 0.099 ± 0.041 ^abc^ | 0.050 ± 0.002 ^c^ | 0.006 ± 0.001 ^ab^ | 0.002 ± 0.000 ^a^ |
| **168 hours** | 0.101 ± 0.058 ^bc^ | 0.023 ± 0.001 ^ab^ | 0.002 ± 0.000 ^a^ | 0.002 ± 0.002 ^a^ | 0.259 ± 0.056 ^d^ | 0.148 ± 0.009 ^c^ | 0.027 ± 0.000 ^ab^ | 0.002 ± 0.000 ^a^ |

**Notes:** Each value represents the mean ± SD (*n=3*) with different superscript letter in the same time point indicate significant statistical differences (Two-way ANOVA, TukeyHSD, p<0.05) using R version 3.4.3

**Tables S9** The effects of copper and pH over 7 days on chlorophyll *a* (10^-3^ ng/cell) of *Desmodesmus* sp. AARLG074

|  | **Mean Chlorophyll *a* ± SD (*n* = 3)** | | | | | | | |
| --- | --- | --- | --- | --- | --- | --- | --- | --- |
| **pH** | **4** | | | | **6** | | | |
| **Cu (mg/l)** | **0** | **2** | **20** | **50** | **0** | **2** | **20** | **50** |
| **0 hour** | 0.479 ± 0.056 ^c^ | 0.196 ± 0.003 ^b^ | 0.057 ± 0.004 ^a^ | 0.029 ± 0.015 ^a^ | 0.518 ± 0.031^c^ | 0.464 ± 0.004 ^c^ | 0.213 ± 0.017 ^b^ | 0.106 ± 0.013 ^a^ |
| **8 hours** | 0.480 ± 0.192 ^bc^ | 0.209 ± 0.086 ^ab^ | 0.055 ± 0.006 ^a^ | 0.002 ± 0.000 ^a^ | 0.536 ± 0.046 ^c^ | 0.560 ± 0.034 ^c^ | 0.515 ± 0.067 ^c^ | 0.104 ± 0.001 ^a^ |
| **16 hours** | 1.042 ± 0.040 ^cd^ | 0.670 ± 0.070 ^bc^ | 0.024± 0.012 ^a^ | 0.041 ± 0.013 ^a^ | 1.544 ± 0.127 ^e^ | 1.419 ± 0.252 ^de^ | 0.702 ± 0.167 ^c^ | 0.168 ± 0.023 ^ab^ |
| **24 hours** | 1.557 ± 0.051 ^c^ | 0.360 ± 0.017 ^ab^ | 0.041 ± 0.003 ^a^ | 0.076 ± 0.005 ^a^ | 1.567 ± 0.064 ^c^ | 1.529 ± 0.259 ^c^ | 0.610 ± 0.090 ^b^ | 0.273 ± 0.026 ^ab^ |
| **72 hours** | 1.745 ± 0.989 ^c^ | 0.663 ± 0.047 ^abc^ | 0.288 ± 0.174 ^ab^ | 0.117 ± 0.008 ^a^ | 1.194 ± 0.103 ^abc^ | 1.462 ± 0.186 ^bc^ | 0.613 ± 0.112 ^abc^ | 0.041 ± 0.009 ^a^ |
| **120 hours** | 2.174 ± 0.496 ^c^ | 0.641 ± 0.068 ^ab^ | 0.166 ± 0.017 ^a^ | 0.506 ± 0.501 ^a^ | 1.450 ± 0.168 ^bc^ | 1.447± 0.236 ^bc^ | 0.140 ± 0.004 ^a^ | 0.028 ± 0.014 ^a^ |
| **168 hours** | 2.004 ± 1.077 ^bcd^ | 0.669 ± 0.053 ^abc^ | 0.023 ± 0.020 ^a^ | 0.256 ± 0.121 ^ab^ | 3.586 ± 0.616 ^d^ | 2.371 ± 0.168 ^cd^ | 0.642 ± 0.047 ^abc^ | 0.096 ± 0.006 ^a^ |

**Notes:** Each value represents the mean ± SD (*n=3*) with different superscript letter in the same time point indicate significant statistical differences (Two-way ANOVA, TukeyHSD, p<0.05) using R version 3.4.3

**Tables S10** The effects of copper and pH over 7 days on chlorophyll *b* (10^-3^ ng/cell) of *Desmodesmus* sp. AARLG074

|  | **Mean Chlorophyll *b* ± SD (*n* = 3)** | | | | | | | |
| --- | --- | --- | --- | --- | --- | --- | --- | --- |
| **pH** | **4** | | | | **6** | | | |
| **Cu (mg/l)** | **0** | **2** | **20** | **50** | **0** | **2** | **20** | **50** |
| **0 hour** | 0.105 ± 0.013 ^ab^ | 0.162 ± 0.020 ^b^ | 0.131 ± 0.032 ^b^ | 0.063 ± 0.004 ^a^ | 0.114 ± 0.007 ^ab^ | 0.172 ± 0.0015 ^b^ | 0.168 ± 0.001 ^b^ | 0.139 ± 0.026 ^b^ |
| **8 hours** | 0.152 ± 0.067 ^bcd^ | 0.247 ± 0.044 ^d^ | 0.095 ± 0.007 ^ab^ | 0.011 ± 0.002 ^a^ | 0.128 ± 0.007 ^bc^ | 0.214 ± 0.018 ^cd^ | 0.216 ± 0.038 ^cd^ | 0.127 ± 0.007 ^bc^ |
| **16 hours** | 0.231 ± 0.059 ^ab^ | 0.379 ± 0.027 ^bc^ | 0.058 ± 0.007 ^a^ | 0.129 ± 0.026 ^a^ | 0.259± 0.018 ^ab^ | 0.388 ± 0.064 ^bc^ | 0.514 ± 0.116 ^c^ | 0.170 ± 0.035 ^a^ |
| **24 hours** | 0.374 ± 0.056 ^bc^ | 0.412 ± 0.004 ^cd^ | 0.078 ± 0.003 ^a^ | 0.022 ± 0.000 ^a^ | 0.424 ± 0.038 ^cd^ | 0.606 ± 0.085 ^d^ | 0.157 ± 0.036 ^ab^ | 0.211 ± 0.075 ^abc^ |
| **72 hours** | 0.338 ± 0.206 ^ab^ | 0.357 ± 0.053 ^ab^ | 0.243 ± 0.082 ^ab^ | 0.169 ± 0.014 ^ab^ | 0.261 ± 0.003 ^ab^ | 0.452 ± 0.077 ^b^ | 0.284 ± 0.089 ^ab^ | 0.100 ± 0.006 ^a^ |
| **120 hours** | 0.485 ± 0.074 ^de^ | 0.352 ± 0.011 ^cd^ | 0.185 ± 0.019 ^ab^ | 0.531± 0.066 ^e^ | 0.547 ± 0.042 ^e^ | 0.293 ± 0.009 ^bc^ | 0.094 ± 0.022 ^a^ | 0.073 ± 0.000 ^a^ |
| **168 hours** | 0.397 ± 0.209 ^a^ | 0.257 ± 0.042 ^a^ | 0.043 ± 0.032 ^a^ | 0.228 ± 0.263 ^a^ | 0.643 ± 0.094 ^a^ | 1.313 ± 1.054 ^a^ | 0.308 ± 0.063 ^a^ | 0.169 ± 0.017 ^a^ |

**Notes:** Each value represents the mean ± SD (*n=3*) with different superscript letter in the same time point indicate significant statistical differences (Two-way ANOVA, TukeyHSD, p<0.05) using R version 3.4.3

**Tables S11** The effects of copper and pH over 7 days on total carotenoids (10^-3^ ng/cell) of *Desmodesmus* sp. AARLG074

|  | **Mean Total Carotenoids ± SD (*n* = 3)** | | | | | | | |
| --- | --- | --- | --- | --- | --- | --- | --- | --- |
| **pH** | **4** | | | | **6** | | | |
| **Cu (mg/l)** | **0** | **2** | **20** | **50** | **0** | **2** | **20** | **50** |
| **0 hour** | 0.122 ± 0.020 ^d^ | 0.053 ± 0.001 ^bc^ | 0.016 ± 0.001 ^a^ | 0.009 ± 0.002 ^a^ | 0.117 ± 0.015 ^d^ | 0.118 ± 0.009 ^d^ | 0.054 ± 0.006 ^c^ | 0.028 ± 0.006 ^ab^ |
| **8 hours** | 0.116 ± 0.056 ^bc^ | 0.056 ± 0.015 ^ab^ | 0.015 ± 0.001 ^a^ | 0.000 ± 0.000 ^a^ | 0.127 ± 0.019 ^bc^ | 0.153 ± 0.020 ^c^ | 0.115 ± 0.014 ^bc^ | 0.033 ± 0.004 ^a^ |
| **16 hours** | 0.264 ± 0.032 ^bc^ | 0.206 ± 0.004 ^ab^ | 0.046 ± 0.005 ^a^ | 0.068 ± 0.008 ^a^ | 0.428 ± 0.051 ^d^ | 0.394 ± 0.079 ^cd^ | 0.143 ± 0.026 ^ab^ | 0.049 ± 0.019 ^a^ |
| **24 hours** | 0.405 ± 0.085 ^bc^ | 0.280 ± 0.021 ^abc^ | 0.045 ± 0.001 ^a^ | 0.078 ± 0.000 ^a^ | 0.435 ± 0.045 ^bc^ | 0.485 ± 0.044 ^c^ | 0.168 ± 0.017 ^a^ | 0.215 ± 0.195 ^ab^ |
| **72 hours** | 0.530 ± 0.302 ^b^ | 0.170 ± 0.021 ^ab^ | 0.175 ± 0.138 ^ab^ | 0.076 ± 0.004 ^ab^ | 0.333 ± 0.030 ^ab^ | 0.391 ± 0.020 ^ab^ | 0.152 ± 0.016 ^ab^ | 0.027 ± 0.003 ^a^ |
| **120 hours** | 0.625 ± 0.220 ^c^ | 0.161 ± 0.004 ^ab^ | 0.088 ± 0.017 ^ab^ | 0.269 ± 0.115 ^ab^ | 0.330 ± 0.004 ^abc^ | 0.375 ± 0.121 ^bc^ | 0.054 ± 0.011 ^ab^ | 0.021 ± 0.002 ^a^ |
| **168 hours** | 0.661 ± 0.404 ^ab^ | 0.208 ± 0.020 ^a^ | 0.025 ± 0.005 ^a^ | 0.047 ± 0.054 ^a^ | 1.233 ± 0.267 ^b^ | 2.731 ± 0.511 ^c^ | 0.324 ± 0.030 ^a^ | 0.038 ± 0.002 ^a^ |

**Notes:** Each value represents the mean ± SD (*n=3*) with different superscript letter in the same time point indicate significant statistical differences (Two-way ANOVA, TukeyHSD, p<0.05) using R version 3.4.3

**Figure 1S**


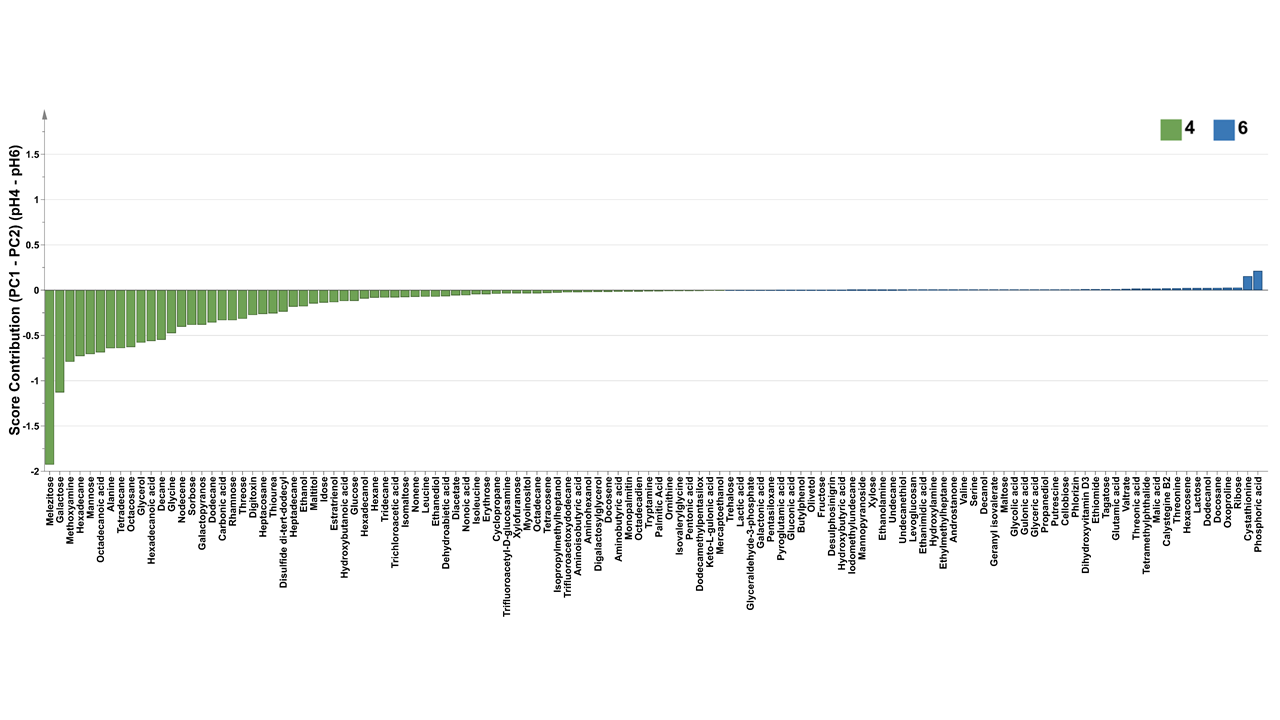


**Figure 1S: The score contribution plot values (full list) which contribute towards GC-MS PCA loading plots of *Desmodesmus* sp. AARLG074 under different pH condition.** The score contribution plot values were ranked in order of importance and are negative if they contribute towards PCA loading plot for the pH4 (green) and positive if they contribute towards the pH6 (blue).

**Figure 2S**


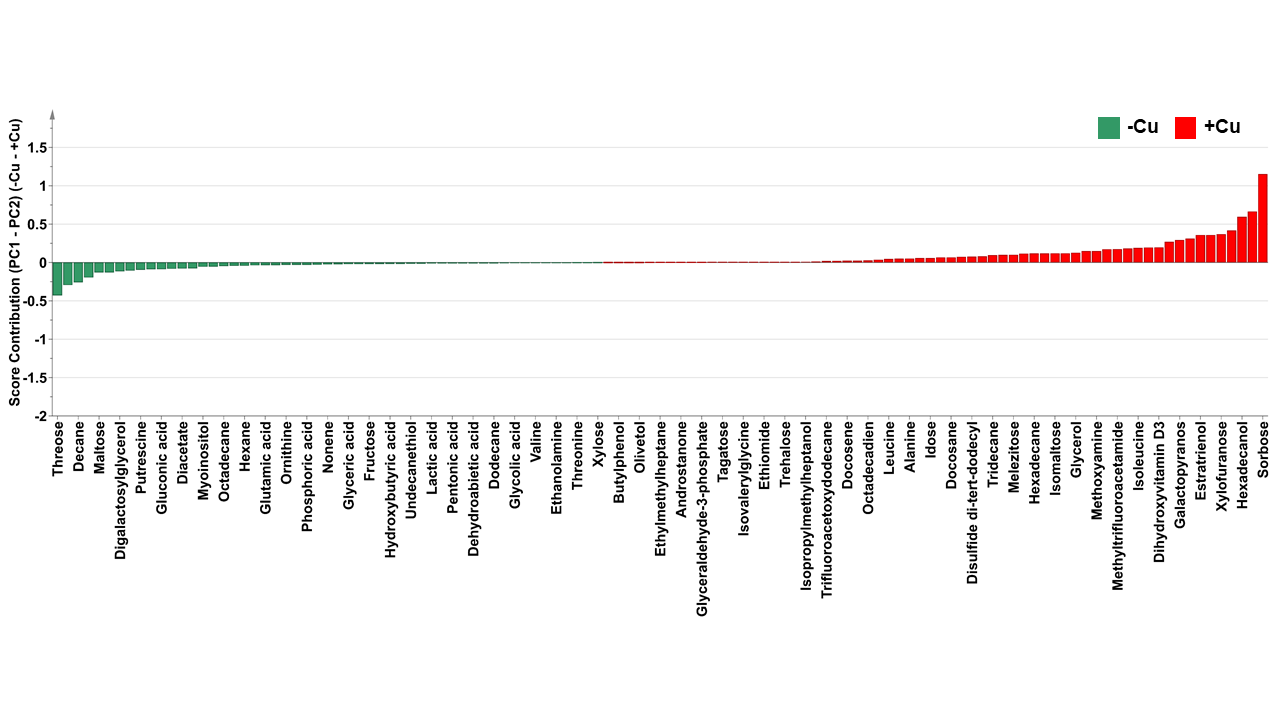


**Figure 2S: The score contribution plot values (full list) which contribute towards GC-MS PCA loading plots of *Desmodesmus* sp. AARLG074 under different pH condition.** The score contribution plot values were ranked in order of importance and are negative if they contribute towards PCA loading plot for the control (blue) and positive if they contribute towards the copper supplemented (red).

**Figure 3S**

**
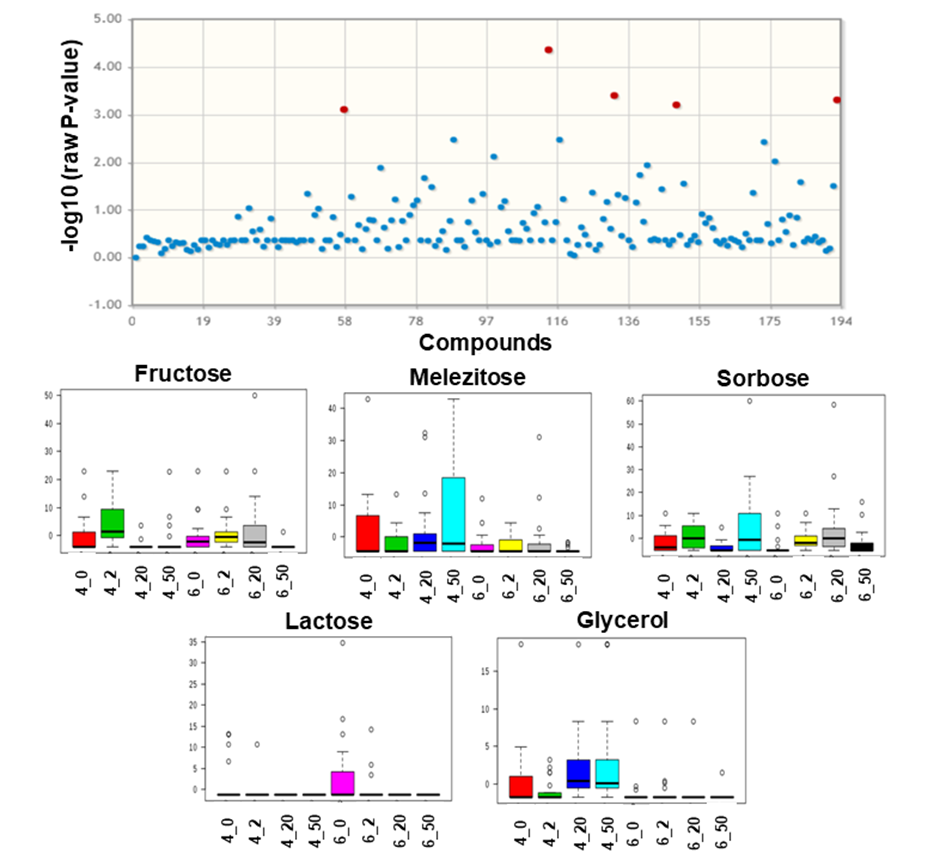
**

**Figure 3S: Statistical analysis of GC-MS metabolite profiling of *Desmodesmus* sp. AARLG074 grown under various copper and pH conditions.** The statistical analysis (One-way ANOVA with Turkey’s LSD) spot represents each metabolite and the red spots are the metabolites that are significantly different (P≤0.05) between treatments. The label on x-axis is the treatments, 4_0, 4_2, 4_20, 4_50 and 6_0, 6_2, 6_20, 6_50 which the first letter is the pH and the second number indicate the copper concentrations. The level of fructose was significantly lower under high copper stress (20 and 50 mg Cu/ L which equal 314.66 and 786.66 µM Cu^2+^ respectively) than control and low copper concentration (2 mg Cu/l)**.** The results show the increase of sorbose under copper and pH stress. Furthermore, pH was a crucial factor on the level of glycerol as there was considerably more glycerol under pH 4 whereas there was no change under pH 6.
