# Supplementary figures and images for: Effects of Copper and pH on the Growth and Physiology of Desmodesmus sp. AARLG074
Source: Metabolites. 2019 Apr 30;9(5):84. doi: 10.3390/metabo9050084 (PMC6572535; doi:10.3390/metabo9050084)

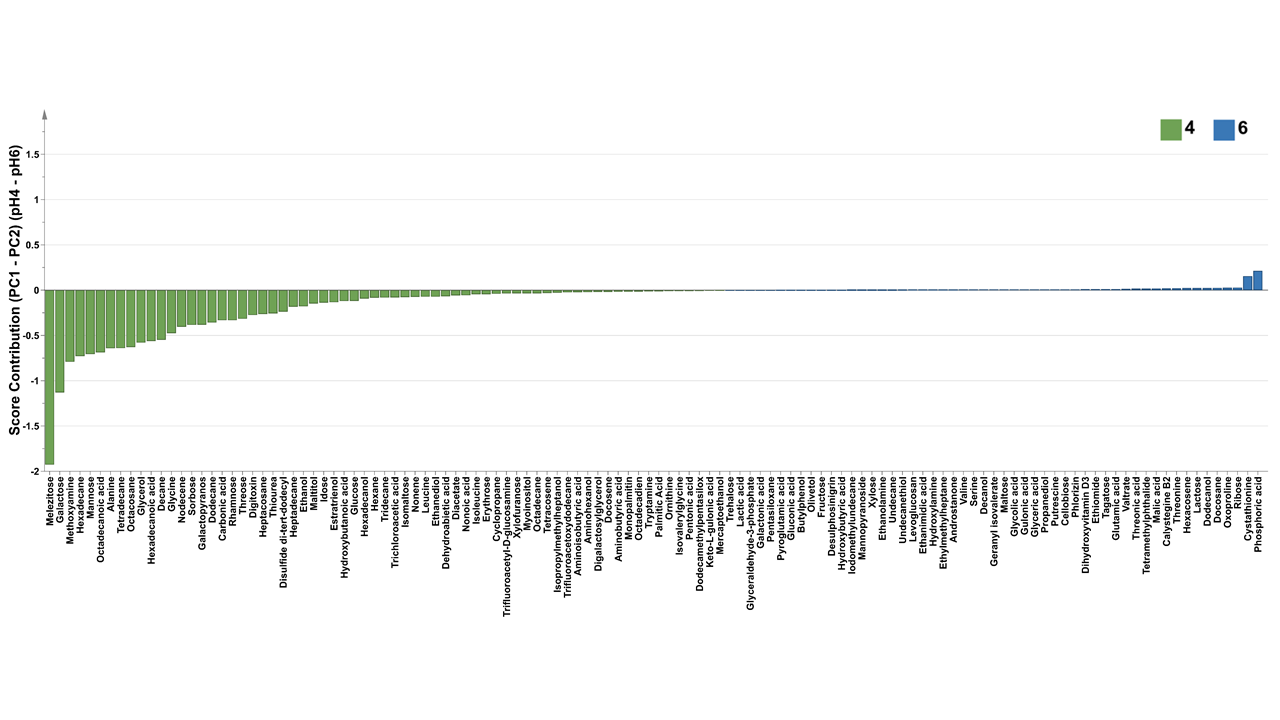

Supplement: Supplementary file 1 [file metabolites-09-00084-s001.zip › Supplementary Materials/Figure S1.TIF]

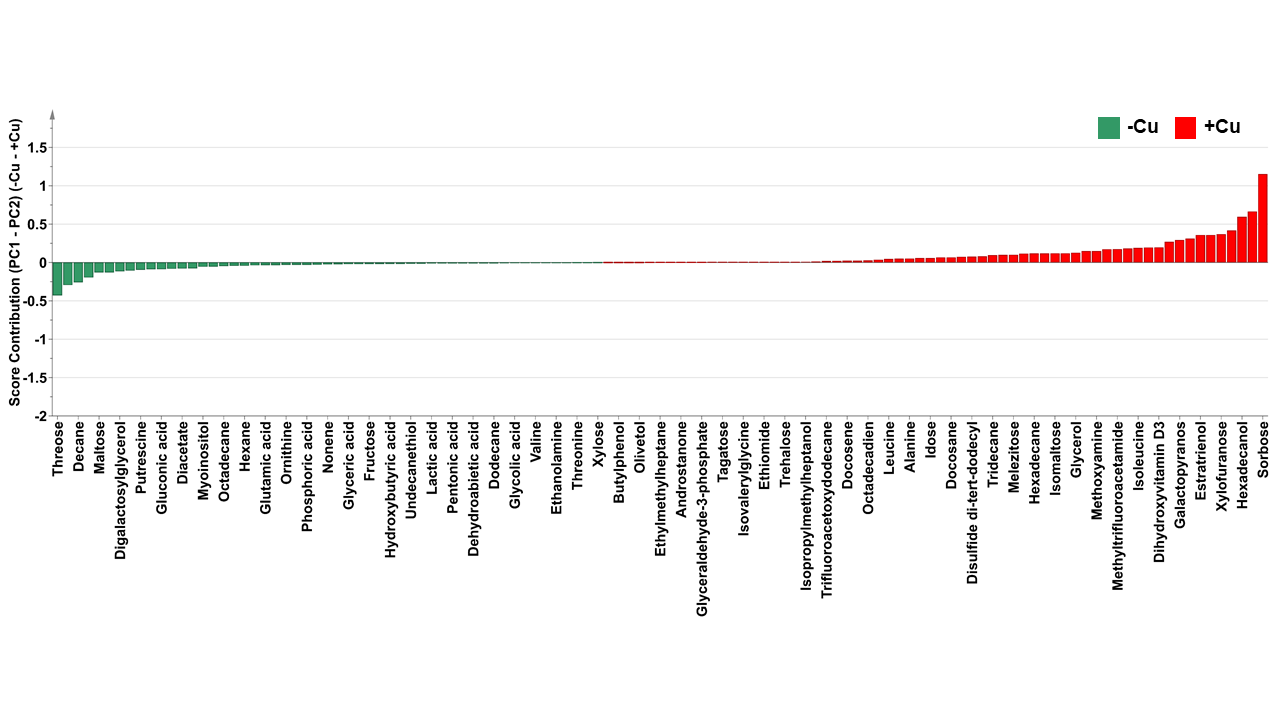

Supplement: Supplementary file 1 [file metabolites-09-00084-s001.zip › Supplementary Materials/Figure S2.TIF]

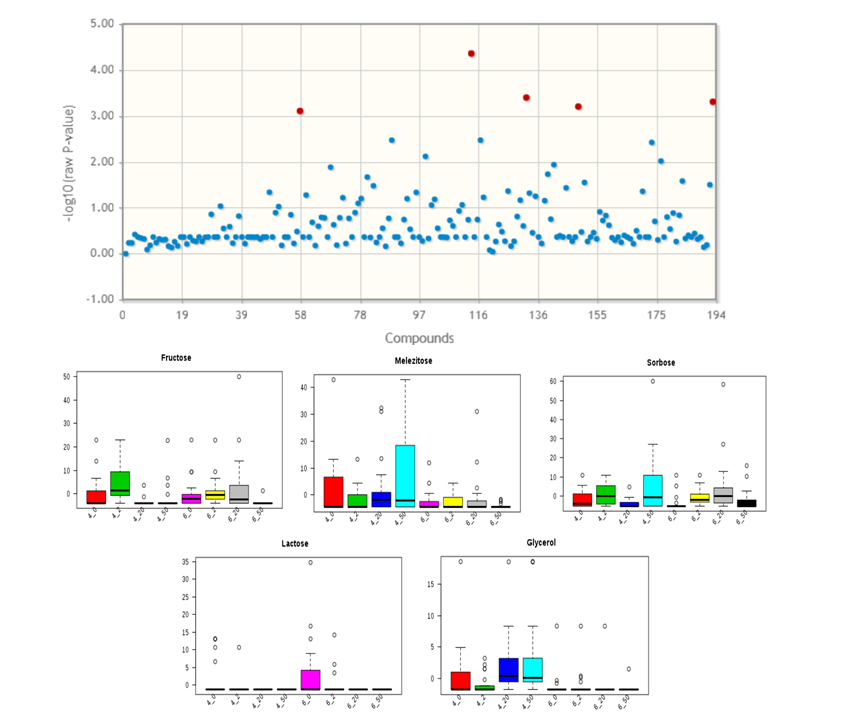

Supplement: Supplementary file 1 [file metabolites-09-00084-s001.zip › Supplementary Materials/Figure S3.tif]
